# Recordings of *Caenorhabditis elegans* locomotor behaviour following targeted ablation of single motorneurons

**DOI:** 10.1038/sdata.2017.156

**Published:** 2017-10-19

**Authors:** Yee Lian Chew, Denise S. Walker, Emma K. Towlson, Petra E. Vértes, Gang Yan, Albert-László Barabási, William R. Schafer

**Affiliations:** 1 Division of Neurobiology, MRC Laboratory of Molecular Biology, Cambridge Biomedical Campus, Francis Crick Avenue, Cambridge CB2 0QH, UK; 2 Center for Complex Network Research and Department of Physics, Northeastern University, Boston, Massachusetts 02115, USA; 3 Department of Psychiatry, Behavioural and Clinical Neuroscience Institute, University of Cambridge, Cambridge CB2 0SZ, UK; 4 School of Physics Science and Engineering, Tongji University, Shanghai 200092, China; 5 Center for Cancer Systems Biology, Dana Farber Cancer Institute, Boston, Massachusetts 02115, USA; 6 Department of Medicine, Brigham and Women’s Hospital, Harvard Medical School, Boston, Massachusetts 02115, USA; 7 Center for Network Science, Central European University, Budapest H-1051, Hungary

**Keywords:** Imaging, Caenorhabditis elegans, Motor control, Behavioural methods

## Abstract

Lesioning studies have provided important insight into the functions of brain regions in humans and other animals. In the nematode *Caenorhabditis elegans*, with a small nervous system of 302 identified neurons, it is possible to generate lesions with single cell resolution and infer the roles of individual neurons in behaviour. Here we present a dataset of ~300 video recordings representing the locomotor behaviour of animals carrying single-cell ablations of 5 different motorneurons. Each file includes a raw video of approximately 27,000 frames; each frame has also been segmented to yield the position, contour, and body curvature of the tracked animal. These recordings can be further analysed using publicly-available software to extract features relevant to behavioural phenotypes. This dataset therefore represents a useful resource for probing the neural basis of behaviour in *C. elegans*, a resource we hope to augment in the future with ablation recordings for additional neurons.

## Background & Summary

The nematode *Caenorhabditis elegans* has become an important experimental organism for neuroscience in part because of its small and well-characterised nervous system. Like many nematodes, the *C. elegans* nervous system is invariant in cell number, consisting of 302 neurons in the adult hermaphrodite. Each of these neurons can be uniquely identified based on its cell lineage and position in the animal, and the connections made by each neuron have been determined by reconstructions of serial electron micrographs^1^. This complete connectome provides an opportunity to address the roles of individual neurons in the generation and control of specific behaviours.

Because individual *C. elegans* neurons can be identified based on the position of their cell body, they can be lesioned with single-cell resolution, providing a powerful tool to investigate the neural basis of behaviour^2^. The most common approach, laser ablation, involves focusing a laser microbeam on a neuronal nucleus, killing the cell but leaving the surrounding cells intact. Cell-specific GFP reporter transgenes are often used to aid in cell identification as well as confirmation of cell killing. Laser ablation experiments have been instrumental in identifying roles for many *C. elegans* neurons. For example, ablations of specific chemosensory neurons identified cells responsible for behavioural responses to specific odorants and soluble repellents^3^, which were subsequently shown to be primary sensory receptors for those molecules.

Cell ablations can also be used to probe the roles of specific neurons in the control of motor behaviour. Motorneurons represent about a third of all *C. elegans* neurons, with 116 neurons (broken down into 22 classes) making neuromuscular junctions with head or body somatic muscle. Ablation experiments in which all or most motorneurons in a class were killed have revealed some functional information about the motor circuitry; for example, ablation of the 6 of the 9 cholinergic DAs or the 5 of 7 cholinergic DBs specifically impairs backward or forward locomotion respectively^4^. Likewise, ablation experiments have identified premotor interneurons whose ablation specifically affects backward or forward locomotion^4^ or large turns^5^. However, despite significant differences in connectivity between motorneurons within a given class, ablation data addressing the consequences of killing single motorneurons have been lacking.

To effectively analyse the effects of single-neuron ablations in the motor circuit of *C. elegans* requires robust approaches for scoring subtle locomotion phenotypes. In recent years, this problem has been addressed through the use of automated imaging and machine vision to analyse worm behaviour. A number of systems have been described for tracking and recording freely-moving *C. elegans*, and then using computational algorithms to quantitatively analyse the locomotor dynamics of the tracked animals^6^. Some studies have classified locomotor behaviour on the basis of features such as speed, curvature, and the onset of reversals and turns^7,8^, while others have used unsupervised approaches to analyse video data more directly^9^. In particular, a seminal study showed that the postural variation in wild-type *C. elegans* can largely be described using a four-dimensional basis called ‘eigenworms’, by which any worm posture can be represented by the relative contributions of four prototypical shapes^10^. As wild-type eigenworms also robustly represent the postures of mutant worms^11^, this allows a compact representation of worm movement for subsequent unsupervised analysis.

**Figure 1:**
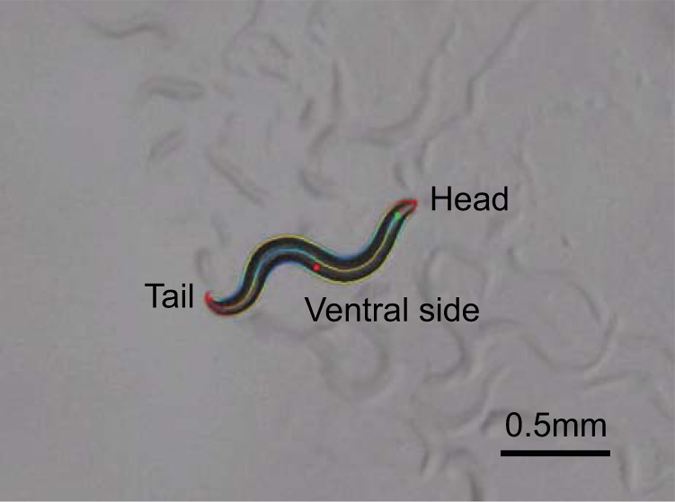
Worm segmentation in tracking videos. For phenotypic analysis, the videos are segmented to extract the worm contour and skeleton. The green dot indicates (on the right side of this image) the position of the head, and the red dot in the midbody indicates the ventral side of the animal. The head and the tail are located as sharp, convex angles on either side of the contour. Head and tail positons are determined automatically. The ventral side of the animal is determined by eye and annotated prior to analysis. For details, see Ref 15.

Here we present a dataset of raw and processed video recordings of *C. elegans* carrying single motorneuron ablations along with mock-ablated control recordings. These include animals lacking one of four DD motorneurons (ventral cord neurons which relax dorsal body muscle) as well as animals lacking the PDB neuron, a cell whose function has not been previously investigated. For each recording, we include the raw recording, accessory data files required for analysis, a processed recording in which frames have been segmented to identify the tracked worm and determine its contour and curvature (Fig. 1), and a feature file containing frame-by-frame measurements of locomotion-related features, including eigenworm data. Although these data were originally collected to test theoretical predictions about neural requirements for motor control^12^, they also represent a useful resource for investigators interested in carrying out their own analyses of nematode locomotion. In the future, we intend to augment this dataset with additional neural ablations, with the long-term goal of comprehensively phenotyping all single neuron ablations in *C. elegans*.

## Methods

### Experimental workflow

The experiments in the dataset (Data Citation 1) were carried out as follows:

The general procedure is as follows:

1. Animals are grown to the stage required for ablation e.g., L1 for DD neurons, L2/early L3 for PDB
2. Animals are either laser-ablated, or mock-ablated (no exposure to laser but still mounted in sodium azide on agarose pads in the same way as laser-ablated animals) and recovered onto bacteria-seeded plates
3. Once animals reach adulthood, they are tracked for 15 min spontaneously moving on bacteria-seeded low peptone plates. The raw video file and accessory files generated during recording are provided in the dataset. We note the position of the ventral side of the animal (Left or Right) in the file name, as well as the neuron ablated and whether the animals were tracked on or off food (all experiments conducted on food in this study). For example, ‘DD2_on food_R’ is the first part of the file name, followed by the date, and the last two numbers represent the number of the tracker (1–8) and the number of the video tracked on that day i.e. for the 10th video obtained from Tracker 8 the last two numbers would be ‘8_10’. Mock-ablated control animals are always tracked at the same time as neuron-ablated animals, and treatment conditions are rotated between trackers to prevent tracker specific effects.
4. Once all videos are obtained, the raw video is segmented (segmented videos provided in dataset where the file name is the same as for the raw video file but with ‘_seg’ at the end) and analysed for features. The segmented images include the contour of the tracked animal as well as a midline skeleton from which body curvature can be computed. The head and tail of the animal are also identified (head indicated by a green dot and a red dot, tail by a red dot only. The red dot at the midbody indicates the ventral side (Fig. 1).
5. In addition to generating the segmented videos, the Worm Tracker script generates a Matlab feature file (provided in the dataset) with the same file name as the raw video file with ‘_features’ at the end. This contains frame-by-frame measurements of various features, including the first six eigenworm projections for each image.
6. This Matlab features file can subsequently be analysed by Matlab scripts (available at http://www.mrc-lmb.cam.ac.uk/wormtracker/, subject to license agreement) into a spreadsheet with summary statistics for the 702 features listed for each video.

Full details are available in Tables 1 and 2.

### Laser killing of C. elegans neurons

Specific neurons were killed by laser ablation, as described previously^2^. DD neurons were killed in L1 animals, using AQ2968 (*ljIs134[Punc-47::GFP-SL2-tagRFP-T]*, a gift from Dr Victoria Butler) as a fluorescent marker. PDB was killed in L2/early L3 animals, since it is born later, using OH904 (*otIs33[kal-1::gfp])*^13^ as a marker. For mock ablations, animals of the same genotype as those to be ablated were mounted and treated with sodium azide in the same way as ablated animals.

### Tracking of ablated and mock-ablated animals

Worms were routinely cultured at room temperature (22 °C) on Nematode Growth Medium (NGM) plates seeded with *E. coli* OP50^14^. Tracking was carried out on 3 cm NGM Low Peptone plates (as standard NGM, except that agar is increased to 2% and peptone is decreased to 0.013%). Plates were stored at 4 °C then dried for approximately 24 h at room temperature before use. On the day of tracking plates were seeded with 20 μl of OP50 overnight culture and allowed to dry. Worms were moved to their tracking plate using a mounted eyelash hair and allowed to acclimatise for 30 min. Ablated animals and mock-ablated controls were tracked together on the same day. The camera magnification was set to between 3.5 and 4.5 μm per pixel with 640×480 video resolution and computer vision software was used to control a motorised stage, thus allowing the camera to follow the worm^15^. For each treatment (ablation or mock ablation), at least 20 day 1 adult hermaphrodites were tracked for 15 min, spontaneously behaving on food.

### Analysis of tracking videos

Videos were segmented and analysed using Worm Tracker 2.0 software^15^. Specifically, for each frame, worm pixels were segmented from the background using the Otsu threshold^16^, the worm being taken as the largest connected component in the resulting image. The curvature of the outline of this connected component was determined, the two points of highest curvature being taken to be the head and the tail. The skeleton was found by tracing the midline of this outline between the head and tail points. The skeleton was then divided into 49 equally spaced points, which were used to define 48 tangent angles and the mean of these angles was then subtracted. A total of 702 features^15^, describing various locomotor parameters, were computed for each video.

With regard to eigenworm features (which track the dynamics of the worm's postural changes by estimating the contributions of prototypical body shapes): the Worm Tracker 2.0 software in its current form does not take into account whether the tracked animal was lying on its left or right side. Thus, in order to detect asymmetries in dorsal versus ventral bending, it is necessary to multiply eigenworm values by −1 for videos in which the animal was on its left side in order to obtain equivalent results to those from right-side recordings. This is particularly important for eigen projection 1 (which corresponds to large body bends), since large turns are normally biased to the ventral side in intact, wild-type animals^5,8^.

In each video, some frames could not be analysed, for example because they were captured during a stage movement, were out of focus, or the worm was in a coiled posture that could not be segmented. In one video (ablPDB on food L_2016_11_03__15_19_19___7___3), most frames could not be segmented; this video was not included in the analysis in Yan *et al.*^12^ and probably represented an injured worm. The proportions of unanalysed frames were (mean±s.d.): DD2/DD5/mock experiment 1, 0.057±0.034; DD2/DD5/mock experiment 2, 0.060±0.041; DD3/DD4/mock experiment 1, 0.061±0.046; PDB/mock experiment 1: 0.059±0.061; PDB/mock experiment 2, 0.048±0.031 (excluding the potentially injured animal described above). Unanalysed frame frequencies for individual movies are included in the metadata record for this repository.

### Code availability

Worm Tracker 2.0 is freely available (subject to license agreement) at http://www.mrc-lmb.cam.ac.uk/wormtracker/. The software license agreement is a non-commercial license for academic use only. For further details, the full license can be found at http://www.mrc-lmb.cam.ac.uk/wormtracker/webcontent/license.pdf.

## Data Records

### Data archive files

The dataset (Data Citation 1) contains 12 archive files in .zip format. When expanded, six of these (those titled ‘raw videos’) contain 35–62 (for exact numbers see methods) video files in.avi format. The other six (those titled ‘feature files and segmented movies) contain 35–62 compressed and segmented videos in .avi format, as well as 35–62 feature files in .mat format. A detailed description of these files follows:

**PDB ablations_1 raw videos (mock/PDB)** (filename PDB ablations_1 raw videos.zip) Raw tracking videos of mock- and PDB-ablated animals freely-moving on a bacterial lawn for 15 min, for replicate 1 of the PDB-ablation experiment.

**PDB ablations_1 feature files and segmented movies** (filename PDB ablations_1 feature files and segmented movies.zip) Processed movies (with segmentation) of mock- and PDB-ablated animals freely-moving on a bacterial lawn for 15 min, and .mat files containing features extracted from analysed videos, for replicate 1 of the PDB-ablation experiment.

**PDB ablations_2 raw videos (mock/PDB)** (filename PDB ablations_2 raw videos.zip) Raw tracking videos of mock- and PDB-ablated animals freely-moving on a bacterial lawn for 15 min, for replicate 2 of the PDB-ablation experiment.

**PDB ablations_2 feature files and segmented movies** (filename PDB ablations_2 feature files and segmented movies.zip) Processed movies (with segmentation) of mock- and PDB-ablated animals freely-moving on a bacterial lawn for 15 min, and .mat files containing features extracted from analysed videos, for replicate 2 of the PDB-ablation experiment.

**DD3 DD4 ablations raw videos (mock/DD3/DD4)** (filename DD3 DD4 ablations_1 raw videos.zip) Raw tracking videos of mock-, DD3-, and DD4-ablated animals freely-moving on a bacterial lawn for 15 min, for the DD3/DD4 ablation experiment.

**DD3 DD4 ablations_1 feature files and segmented movies** (filename DD3 DD4 ablations_1 feature files and segmented movies.zip) Processed movies (with segmentation) of mock- and ablated animals freely-moving on a bacterial lawn for 15 min, and .mat files containing features extracted from analysed videos, for the DD3/DD4-ablation experiment.

**DD2 DD5 ablations_2 raw videos (mock)** (filename DD2 DD5 ablations_2 raw videos mock.zip) Raw tracking videos of mock-ablated animals freely-moving on a bacterial lawn for 15 min, for replicate 2 of the DD2/DD5 ablation experiment.

**DD2 DD5 ablations_2 raw videos (DD2/DD5)** (filename DD2 DD5 ablations_2 raw videos DD2 DD5.zip) Raw tracking videos of DD2-, and DD5-ablated animals freely-moving on a bacterial lawn for 15 min, for replicate 2 of the DD2/DD5 ablation experiment.

**DD2 DD5 ablations_2 feature files and segmented movies** (filename DD2 DD5 ablations_2 feature files and segmented movies.zip) Processed movies (with segmentation) of mock- and DD2- and DD5-ablated animals freely-moving on a bacterial lawn for 15 min, and .mat files containing features extracted from analysed videos, for replicate 2 of the DD2/DD5-ablation experiment.

**DD2 DD5 ablations_1 raw videos (mock)** (filename DD2 DD5 ablations_1 raw videos mock.zip) Raw tracking videos of mock-ablated animals freely-moving on a bacterial lawn for 15 min, for replicate 1 of the DD2/DD5 ablation experiment.

**DD2 DD5 ablations_1 raw videos (DD2/DD5)** (filename DD2 DD5 ablations_1 raw videos DD2DD5.zip) Raw tracking videos of DD2- and DD5-ablated animals freely-moving on a bacterial lawn for 15 min, for replicate 1 of the DD2/DD5 ablation experiment.

**DD2 DD5 ablations_1 feature files and segmented movies** (filename DD2 DD5 ablations_1 feature files and segmented movies.zip) Processed movies (with segmentation) of mock- and DD2- and DD5-ablated animals freely-moving on a bacterial lawn for 15 min, and .mat files containing features extracted from analysed videos, for replicate 1 of the DD2/DD5-ablation experiment.

### Accessory data files

We have also included accessory files (log.csv, info.xml, info.xml.vignette.dat) containing metadata that allow analysis of the raw videos using our Worm Analysis Toolbox. One compressed folder contains five subfolders named as follows:

**Data files DD2 DD5 1:** accessory files for the videos of DD2- and DD5-ablated animals and corresponding mock-ablated controls, for replicate 1 of the DD2/DD5-ablation experiment.

**Data files DD2 DD5 2:** accessory files for the videos of DD2- and DD5-ablated animals and corresponding mock-ablated controls, for replicate 2 of the DD2/DD5-ablation experiment.

**Data files DD3 DD4:** accessory files for the videos of DD3- and DD4-ablated animals and corresponding mock-ablated controls, for the DD3/DD4-ablation experiment.

**Data files PDB 1:** accessory files for the videos of PDB-ablated animals and corresponding mock-ablated controls, for replicate 1 of the PDB-ablation experiment.

**Data files PDB 2:** accessory files for the videos of PDB-ablated animals and corresponding mock-ablated controls, for replicate 2 of the PDB-ablation experiment.

### Sample video files

Also included are six sample videos, representative of the segmented video files in the data archives. The DD-ablated (and mock-ablated control) movies are all from the ‘DD2 DD5 ablations_2 feature files and segmented movies’ archive. The PDB-ablated (and mock-ablated control) movies are all from the ‘PDB ablations_2 feature files and segmented movies’ archive. The following files are included:

**Mock-ablation (DD) sample video**

(filename mockDD_onfood_L_2016_10_29__13_13_35___7___6_seg.avi)

**DD5-ablation sample video**

(filename DD5_onfood_L_2016_10_29__13_13_25___5___6_seg.avi)

**DD2-ablation sample video**

(filename DD2_onfood_R_2016_10_30__12_13_57___7___4_seg.avi)

**Mock-ablation (PDB) sample video**

(filename mockPDB on food L_2016_11_03__14_16_37___7___1_seg.avi)

**PDB-ablation sample video 1**

(filename ablPDB on food L_2016_11_03__14_40_04___4___2_seg.avi)

**PDB-ablation sample video 2**

(filename ablPDB on food L_2016_11_04__14_28_26___5___1_seg.avi)

These files are available under a CC0 waiver (Data Citation 1).

## Technical Validation

In order to remove any bias resulting from heterogeneity in laboratory conditions, media and culture of experimental subjects, the following procedures were followed: Ablated animals were compared with unablated controls, which were genotypically identical. Ablated and unablated animals were cultured together until ablation and were treated identically (e.g., mounted on an agarose pad and incubated in sodium azide) except that the relevant neuron was/was not ablated. Culture plates were prepared, seeded and stored in an identical manner, and care was taken that, where several batches were used, these were equally distributed between treatments. As described in Methods, tracking plates were prepared and seeded following an identical protocol, to minimise heterogeneity in moisture content and food quality and quantity; animals were carefully staged as day 1 adults, and were picked and acclimatised in a standard manner. Room temperature was controlled at 22 °C. To avoid potential bias resulting from heterogeneity in room conditions (temperature, due to uneven air flow; humidity; potential changes in odour input, etc.), recording for each replicate was randomised across multiple trackers and across multiple days, with ablated and unablated animals equally distributed between days and across time points on each day, such that a representative from each treatment was always recorded at the same time as representatives from all other treatments within that experiment.

## Additional Information

**How to cite this article:** Chew, Y. L. *et al.* Recordings of *Caenorhabditis elegans* locomotor behaviour following targeted ablation of single motorneurons. *Sci. Data* 4:170156 doi: 10.1038/sdata.2017.156 (2017).

**Publisher’s note:** Springer Nature remains neutral with regard to jurisdictional claims in published maps and institutional affiliations.

## Supplementary Material



## Figures and Tables

**Table 1:** Genotype and sample size for experimental groups tested in all assays.

**Experiment**	**Genotype**	**Sample size (Replicate 1)**	**Sample size (Replicate 2)**
DD2/DD5 ablation	AQ2968 *(ljIS134[Punc-47::GFP-SL2-tagRFP-T])*	Mock=31DD2=23DD5=26	Mock=27DD2=29DD5=22
DD3/DD4 ablation	AQ2968 *(ljIS134[Punc-47::GFP-SL2-tagRFP-T])*	Mock=23DD3=21DD4=18	
PDB ablation	OH904 *(otIS33[kal-1::gfp])*	Mock=19PDB=24	Mock=16PDB=19

**Table 2:** Description of experimental conditions and samples tested.

**Strain name**	**Organism**	**Reporter line**	**Method**	**Sample Name**	**Genotype**	**Treatment**
AQ2968 *(ljIS134[Punc-47::GFP-SL2-tagRFP-T])*	*Caenorhabditis elegans*	Transgenic reporter line used to identify DD neurons	**Laser killing of** ***C. elegans *****neurons** (Mock) followed by **Tracking of ablated and mock-ablated animals**	Mock-ablated DD	*ljIS134[Punc-47::GFP-SL2-tagRFP-T]*	Animals were mounted in sodium azide on an agarose pad, no laser ablation
AQ2968 *(ljIS134[Punc-47::GFP-SL2-tagRFP-T])*	*Caenorhabditis elegans*	Transgenic reporter line used to identify DD neurons	**Laser killing of** ***C. elegans *****neurons** (DD2) followed by **Tracking of ablated and mock-ablated animals**	DD2-ablated	*ljIS134[Punc-47::GFP-SL2-tagRFP-T]*	Animals were mounted in sodium azide on an agarose pad, laser ablation of DD2
AQ2968 *(ljIS134[Punc-47::GFP-SL2-tagRFP-T])*	*Caenorhabditis elegans*	Transgenic reporter line used to identify DD neurons	**Laser killing of** ***C. elegans *****neurons** (DD5) followed by **Tracking of ablated and mock-ablated animals**	DD5-ablated	*ljIS134[Punc-47::GFP-SL2-tagRFP-T]*	Animals were mounted in sodium azide on an agarose pad, laser ablation of DD5
AQ2968 *(ljIS134[Punc-47::GFP-SL2-tagRFP-T])*	*Caenorhabditis elegans*	Transgenic reporter line used to identify DD neurons	**Laser killing of** ***C. elegans *****neurons** (DD3) followed by **Tracking of ablated and mock-ablated animals**	DD3-ablated	*ljIS134[Punc-47::GFP-SL2-tagRFP-T]*	Animals were mounted in sodium azide on an agarose pad, laser ablation of DD3
AQ2968 *(ljIS134[Punc-47::GFP-SL2-tagRFP-T])*	*Caenorhabditis elegans*	Transgenic reporter line used to identify DD neurons	**Laser killing of** ***C. elegans *****neurons** (DD4) followed by **Tracking of ablated and mock-ablated animals**	DD4-ablated	*ljIS134[Punc-47::GFP-SL2-tagRFP-T]*	Animals were mounted in sodium azide on an agarose pad, laser ablation of DD4
OH904 *(otIS33[kal-1::gfp])*	*Caenorhabditis elegans*	Transgenic reporter line used to identify PDB neuron	**Laser killing of** ***C. elegans *****neurons** (Mock) followed by **Tracking of ablated and mock-ablated animals**	Mock-ablated PDB	*otIS33[kal-1::gfp]*	Animals were mounted in sodium azide on an agarose pad, no laser ablation
OH904 *(otIS33[kal-1::gfp])*	*Caenorhabditis elegans*	Transgenic reporter line used to identify PDB neuron	**Laser killing of** ***C. elegans *****neurons** (PDB) followed by **Tracking of ablated and mock-ablated animals**	PDB-ablated	*otIS33[kal-1::gfp]*	Animals were mounted in sodium azide on an agarose pad, laser ablation of PDB
For each recorded animal, the experimental details (genotype, lesion, treatment) are described.						
